# The Synthesis of New Thermal Stable Schiff Base/Ester Liquid Crystals: A Computational, Mesomorphic, and Optical Study

**DOI:** 10.3390/molecules24173032

**Published:** 2019-08-21

**Authors:** Sherif S. Nafee, Mohamed Hagar, Hoda A. Ahmed, Reda M. El-Shishtawy, Bahaaudin M. Raffah

**Affiliations:** 1 Physics Department, Faculty of Science, King Abdulaziz University, Jeddah 21589, Saudi Arabia; 2 Chemistry Department, College of Sciences, Taibah University, Yanbu 30799, Saudi Arabia; 3 Chemistry Department, Faculty of Science, Alexandria University, Alexandria 21321, Egypt; 4 Department of Chemistry, Faculty of Science, Cairo University, Cairo 12613, Egypt; 5 Department of Chemistry, Faculty of Science, King Abdulaziz University, Jeddah 21589, Saudi Arabia; 6 Dyeing, Printing and Textile Auxiliaries Department, Textile Research Division, National Research Center, Dokki, Cairo 12622, Egypt

**Keywords:** Schiff base liquid crystals, mesomorphic, optical behavior, geometrical parameters, density functional theory (DFT)

## Abstract

A Schiff base supramolecular 4-[(4-(hexyloxy)phenylimino)methyl]benzoic acid and a new series of Schiff base/ester linkages named 4-substitutedphenyl 4-[(4-(hexyloxy)phenylimino)methyl]benzoate liquid crystals were synthesized. The thermal stability, mesomorphic, and optical behavior of the prepared compounds were characterized by differential scanning calorimetry (DSC), Thermogravemetric analysis (TGA), polarized optical microscopy (POM), and UV spectroscopy. FT-IR, ^1^H-NMR, ^13^C-NMR, and elemental analyses were carried out to elucidate and confirm the molecular structures of the synthesized compounds. The investigated series comprising different sized terminal polar groups changed between CH(CH_3_)_2_, H, I, and F. It was found that the supramolecular imino acid dimer is enantiotropic dimorphic, with a wide SmA phase and a good N phase range. The other series of terminally substituted Schiff base/esters are mesomorphic with a high thermal stable SmA phase, except the iodo derivative, which showed dimorphic SmA and N phases. The effect of the position and the orientation of the cores, as well as the terminal substituent of the type and the stability of the mesophase, were studied. A computational theoretical study of the effects of the van der Waal’s volume, the Hammett substituent coefficient, the inductive sigma constant, and other geometrical parameters were discussed. The study revealed that the planarity of the two phenyl rings attached with an imino linking group impacted the resonance effect of the terminal substituents rather than their inductive effect. A detailed study on the effect of the estimated thermal parameters, as well as their geometrical planarity with the type and stability of the formed mesophase, was discussed.

## 1. Introduction

Schiff bases (–CH=N–) have wide application interests as corrosion inhibitors [1], biological active materials [2], and thermo-stable systems [3,4,5]. The optical and semiconducting phenomena of the azomethine linkage group have been also widely investigated due to their photo-efficiency, with wavelengths depending on the chemical architecture of the Schiff-base molecules [6,7]. Generally, multiple bond linkages that maintain the linearity and rigidity of the molecular structure are satisfied in promoting the thermal stability of the mesophases. However, the ester linking unit contains no multiple bonds in the chain of atoms linking the two benzene rings, and conjugative interactions within the ester moiety and the rings yield some double bond characteristics. Hence, the mesophase becomes more persistent when the phase stability effect of the mutual conjugation between the substituent and the ester carbonyl or oxygen is increased. The mesomorphic stability of organic compounds depends primarily on its structural geometry, in which a slight change in the molecular conformation enables are markable change in its mesomeric characteristics [8]. As the polarity and/or polarizability of the molecular core increase, the stability of the formed mesophase increases. Most studies have been focused on Schiff bases ever since the discovery of a room temperature nematic phase of 4-methoxybenzylidene-4′-butylaniline [9]. Several Schiff base mesogenic homologous series of a low molar mass and twist-bend nematic phase have been investigated [10,11,12]. Many of these studies have reported to show the effect of the terminal substituent with either alkoxy chains or polar compact substituents [13]. The proper selection of a mesogenic moiety, terminal groups, and flexible wings are the essential criteria for designing new thermotropic liquid crystal materials with new phase transitions [14]. Interestingly, computational investigation is an excellent tool in designing new materials and has attracted the attention of many researchers [15,16,17,18,19,20,21,22,23]. Moreover, to prepare compounds with proper optical characteristics, it is necessary to stimulate important properties, such as molecular orbital energies, the frontier molecular orbitals energy difference, and the molecular geometries of the investigated liquid crystalline materials. Generally, density functional theory (DFT) proves an effective tool for these kinds of predictions due to its excellent performance and consistent results.

In our previous work [16,24], a series of substituted Schiff base/esters were prepared, and we investigated the effect of the alkoxy chain lengths and the polarity of small, compact terminal groups on their mesophase stability. To further our interests and improve the mesomorphic and photo-physical properties of liquid crystalline materials, supramolecular 4-[(4-(hexyloxy)phenylimino)methyl]benzoic acid (**1**) and 4-substitutedphenyl 4-[(4-(hexyloxy)phenylimino)methyl]benzoate (**A**–**D**) were synthesized and analyzed for their mesophase formation and stability. The present Schiff base/ester series are different from previous homologues **E** and **F** [16,24] in the location and the orientation of the ester linkage and the C=N linkage, as well as in the exchange of the position of terminal substituents. Moreover, detailed theoretical investigations of the effects of van der Waal’s volume, Hammett substituent coefficient, and the inductive sigma constant, as well as the geometrical effects, were studied. A comparative study between the terminally neat derivative and its isomers (positional and orientational of the mesogenic cores, COO, C=N) were investigated to illustrate the effect of the position and the orientation of the cores, as well as the terminal substituent in the type and the stability of the mesophase.


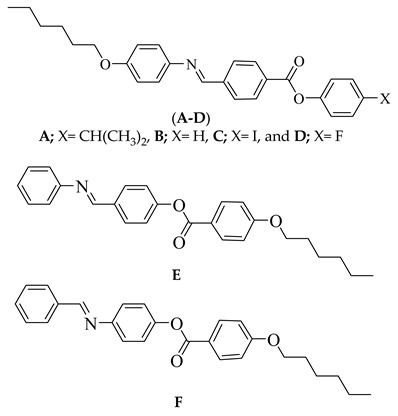


## 2. Experimental

### 2.1. Materials

4-Isopropylphenol, 4-iodophenol, 4-fluorophenol and phenol were purchased from Sigma Aldrich (Hamburg, Germany). Dichloromethane (DCM), *N,N’*-dicyclohexylcarbodiimide (DCC), ethanol and 4-dimethylaminopyridine (DMAP) were purchased from Aldrich (St. Louis, MO, USA). 

### 2.2. Synthesis

**Scheme 1:**
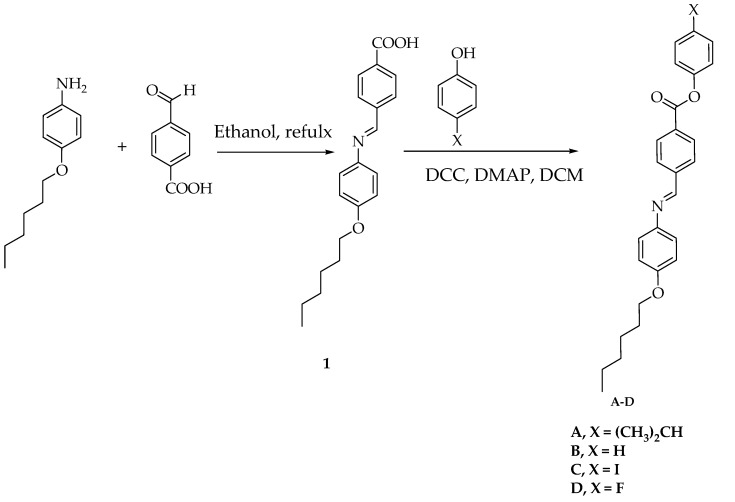
Synthesis of 4-substitutedphenyl 4-[(4-(hexyloxy)phenylimino)methyl]benzoate (**A**–**D**).

Compounds **A**–**D** were prepared according to the following Scheme 1:

#### Synthesis of 4-[(4-(hexyloxy)phenylimino)methyl]benzoic acid (**1**) 

Equimolar amount of 4-formylbenzoic acid (610 mg, 4.1 mmol) and 4-hexyloxyaniline (790 mg, 4.1 mmol) in ethanol (10 mL) were refluxed for two hours. The reaction mixture was allowed to cool, and the separated product was filtered. The obtained solid was recrystallized from ethanol [25,26]. 

Yield: 93.0%; m.p. 190.0 °C, FTIR (ύ, cm^−1^): 2930–2864 (CH_2_ stretching), 1678 (C=O), 1616 (C=N),1597 (C=C), 1490 (C–O_Asym_), 1235 (C–O _Sym_). ^1^H NMR (400 MHz, CDCl_3_) δ 10.03 (s, 1H, CH=N), 8.13 (d, *J* = 8.5 Hz, 2H, ArH), 7.97 (d, *J* = 8.5 Hz, 2H, ArH), 7.40 (d, *J* = 8.4 Hz, 2H, ArH), 6.99 (d, *J* = 8.6 Hz, 2H, ArH), 4.05 (t, *J* = 6.5 Hz, 2H, CH_3_(CH_2_)_3_CH_2_CH_2_), 1.91–1.76 (m, 2H,CH_3_(CH_2_)_3_CH_2_CH_2_), 1.58–1.56 (m, 6H, CH_3_(CH_2_)_3_CH_2_CH_2_), 0.88 (t, *J* = 6.6 Hz, 3H, CH_3_(CH_2_)_3_CH_2_CH_2_). ^13^C NMR (101 MHz, CDCl_3_) δ 191.62, 169.63, 159.57, 139.62, 134.29, 130.78, 129.60, 124.37, 115.44, 115.42, 68.39, 31.55, 29.22, 25.64, 22.60, 14.05. Elemental analyses: Found (Calc.): C, 73.79 (73.82); H, 7.11 (7.12); N, 4.29 (4.30).

#### Synthesis of 4-substitutedphenyl 4-[(4-(hexyloxy)phenylimino)methyl]benzoate (**A**–**D**)

Molar equivalents of 4-[(4-(hexyloxy)phenylimino)methyl] benzoic acid and 4-substitutedphenol (0.01 mole) were dissolved in dry methylene chloride (DCM) (25 mL). 0.02 molar of *N*, *N′*-dicyclohexylcarbodiimide (DCC), and trace amounts of 4-dimethylaminopyridine (DMAP) were added to the reaction mixture. The reaction was left under stirring at room temperature for 72 H. The separated byproduct, dicyclohexylurea (DCU), was filtered off. The filtrate was then evaporated, and the obtained product was recrystallized from ethanol.

#### 4-Isopropylphenyl 4-[(4-(hexyloxy)phenylimino)methyl]benzoate (**A**)

Yield: 87.0%; m.p. 92.0 °C, FTIR (ύ, cm^−1^): 2930–2864 (CH_2_ stretching), 1729 (C=O), 1613 (C=N), 1577 (C=C), 1494 (C–O_Asym_), 1246 (C-O _Sym_).). ^1^H NMR (400 MHz, CDCl_3_) δ 8.57 (s, 0.5H, CH=N), 8.53 (s, 0.5H, CH=N), 8.28 (d, *J* = 8.4 Hz, 1H, ArH), 8.05–7.97 (m, 2H, ArH), 7.75–7.73 (m, 2H, ArH), 7.35–7.22 (m, 3H, ArH), 7.06–7.00 (m, 2H, ArH), 6.95–6.85 (m, 2H, ArH), 4.04 (dt, *J* = 6.4, 2.5 Hz, 2H, CH_3_(CH_2_)_3_CH_2_CH_2_), 2.61 (dd, *J* = 6.3, 2.2 Hz, 1H, CH(CH_3_)_2_), 1.84–1.76 (m, 2H,CH_3_(CH_2_)_3_CH_2_CH_2_), 1.58–0.96 (m, 12H, CH_3_(CH_2_)_3_CH_2_CH_2_, CH(CH_3_)_2_), 0.92 (dt, *J* = 6.4, 4.1 Hz, 3H, CH_3_(CH_2_)_3_CH_2_CH_2_). Elemental analyses: Found (Calc.): C, 78.51 (78.52); H, 7.48 (7.50); N, 3.14 (3.16).

#### 4-Phenyl 4-[(4-(hexyloxy)phenylimino)methyl]benzoate (**B**)

Yield: 91.0%; m.p. 67.0 °C, FTIR (ύ, cm^−1^): 2927–2860 (CH_2_ stretching), 1730 (C=O), 1612 (C=N),1579 (C=C), 1490 (C–O_Asym_), 1244 (C-O _Sym_). ^1^H NMR (400 MHz, CDCl_3_) δ 8.58 (s, 0.4H, CH=N), 8.52 (s, 0.6H, CH=N), 8.24 (d, *J* = 8.5 Hz, 1H, ArH), 8.04–7.91 (m, 2H, ArH), 7.73 (d, *J* = 8.4 Hz, 1H, ArH), 7.52–7.43 (m, 2H, ArH), 7.39–7.23 (m, 5H, ArH), 7.04–7.91 (m, 2H, ArH), 4.00 (dt, *J* = 6.8, 2.4 Hz, 2H, CH_3_(CH_2_)_3_CH_2_CH_2_), 1.95–1.61 (m, 2H,CH_3_(CH_2_)_3_CH_2_CH_2_), 1.59–1.08 (m, 6H, CH_3_(CH_2_)_3_CH_2_CH_2_), 0.93 (dt, *J* = 6.0, 4.0 Hz, 3H, CH_3_(CH_2_)_3_CH_2_CH_2_). Elemental analyses: Found (Calc.): C, 77.76 (77.78); H, 6.78 (6.78); N, 3.47 (3.49).

#### 4-Iodophenyl 4-[(4-(hexyloxy)phenylimino)methyl]benzoate (**C**)

Yield: 92.0%; m.p. 75.0 °C, FTIR (ύ, cm^−1^): FTIR (ύ, cm^−1^): 2930-2864 (CH_2_ stretching), 1728 (C=O), 1613 (C=N),1577 (C=C), 1493 (C–O_Asym_), 1247 (C-O _Sym_).). ^1^H NMR (400 MHz, CDCl_3_) δ 8.58 (s, 0.6H, CH=N), 8.52 (s, 0.6H, CH=N), 8.27 (d, *J* = 8.4 Hz, 1H, ArH), 8.03–7.98 (m, 2H, ArH), 7.77–7.73 (m, 2H, ArH), 7.32–7.24 (m, 3H, ArH), 7.08–7.00 (m, 2H, ArH),6.97–6.88 (m, 2H, ArH),4.00 (dt, *J* = 6.8, 2.4 Hz, 2H, CH_3_(CH_2_)_3_CH_2_CH_2_), 1.84–1.76 (m, 2H,CH_3_(CH_2_)_3_CH_2_CH_2_), 1.58–1.16 (m, 6H, CH_3_(CH_2_)_3_CH_2_CH_2_), 0.86 (dt, *J* = 6.8, 4.0 Hz, 3H, CH_3_(CH_2_)_3_CH_2_CH_2_). Elemental analyses: Found (Calc.): C, 59.21 (59.21); H, 4.95 (4.97); N, 2.65 (2.66); I, 24.04 (24.06).

#### 4-Fluorophenyl 4-[(4-(hexyloxy)phenylimino)methyl]benzoate (**D**)

Yield: 94.0%; m.p. 74.0 °C, FTIR (ύ, cm^−1^): 2928-2863 (CH_2_ stretching), 1729 (C=O), 1611 (C=N), 1582 (C=C), 1496 (C–O_Asym_), 1240 (C-O _Sym_).). ^1^H NMR (400 MHz, CDCl_3_) δ 8.58 (s, 0.4H, CH=N), 8.52 (s, 0.6H, CH=N), 8.28 (dd, *J* = 14.4, 8.8 Hz, 1H, ArH), 8.04–7.91 (m, 2H, ArH), 7.73 (d, *J* = 8.4 Hz, 1H, ArH), 7.32–7.28 (m, 2H, ArH), 7.24–7.18 (m, 2H, ArH), 7.15–7.09 (m, 2H, ArH), 6.97–6.93 (m, 2H, ArH), 4.05 (dt, *J* = 6.4, 2.4 Hz, 2H, CH_3_(CH_2_)_3_CH_2_CH_2_), 1.84–1.76 (m, 2H,CH_3_(CH_2_)_3_CH_2_CH_2_), 1.58–1.16 (m, 6H, CH_3_(CH_2_)_3_CH_2_CH_2_), 0.91 (dt, *J* = 5.6, 2.1 Hz, 3H, CH_3_(CH_2_)_3_CH_2_CH_2_). Elemental analyses: Found (Calc.): C, 74.43 (74.44); H, 6.23 (6.25); N, 3.33 (3.34); F, 4.52 (4.53).

### 2.3. Characterization

The NMR spectra were measured on a Varian EM 350L 300 MHz spectrometer (company, Oxford, UK)), while the elemental analysis (Thermo Scientific Flash 2000 CHS/O Elemental Analyzer, Milan, Italy). Thermogravemetric analysis (TGA) was carried out using a Shimadzu TGA-50H Thermal Analyzer. A Differential Scanning Calorimeter, TA instrument Co. Q20 (DSC; USA), was used for calorimetric measurements. The types of the mesophase texture were identified by a standard polarized optical microscope (POM, Wild, Germany). The UV-Vis spectra were recorded by a UV-1800 (SHIMADZU, Kyoto, Japan) (for detailed specifications, see supplementary data).

### 2.4. Computational Method

Gaussian 09 software was used for DFT calculations of the studied compounds [27]. DFT/B3LYP methods using the LANL2DZ basis set [28] were selected for the calculations. The geometries were optimized by minimizing the energies with respect to all geometrical parameters without imposing any molecular symmetry constraints. The structures of the optimized geometries were drawn with the Gauss View [29]. Further, calculation frequencies were carried out with the same level of theory. The frequency calculations showed that all structures were stationary points in the geometry optimization method, with no imaginary frequencies. 

## 3. Results and Discussion

### 3.1. Infrared Absorption Spectra of Components ***A**–**F***

The IR spectra were measured by Perkin-Elmer B25 spectrophotometer (Perkin-Elmer, Inc., Shelton, CT, USA). The position and the orientation of the mesogenic core have an observable effect on the wavenumber of the absorbance bands of the characteristic peaks for the C=O and C=N of the investigated compounds, **A**–**F**. However, the electronic nature of the terminal substituent has no significant effect on the absorbance wavenumber of these characteristic peaks. This result could be explained in terms of the isolation of the terminal substituent from these groups. The terminally neat derivative (**B**) showed an absorbance band at 1730 cm^−1^ corresponding to the C=O group, which agrees with previous reports [25,26]. The absorbance band of its isomers, **E** and **F**, was 1720 cm^−1^. However, only 4 cm^−1^ are different in the absorption peaks of wavenumbers C=N, 1613 and 1609 cm^−1^ for **B** and **E**, respectively.

Obviously, from the NMR spectroscopy, the reaction effectively proceeded to afford a mixture of two geometrical isomers (**E** and **Z**), but their ratio depends insignificantly on the electronic nature of the terminal substituent (see supplementary data Figures S2–S6). The investigation of such effects on the stability of the obtained isomers could be an important subject for future analyses, either theoretical or experimental.

### 3.2. Mesomorphic Study

**Figure 1:**
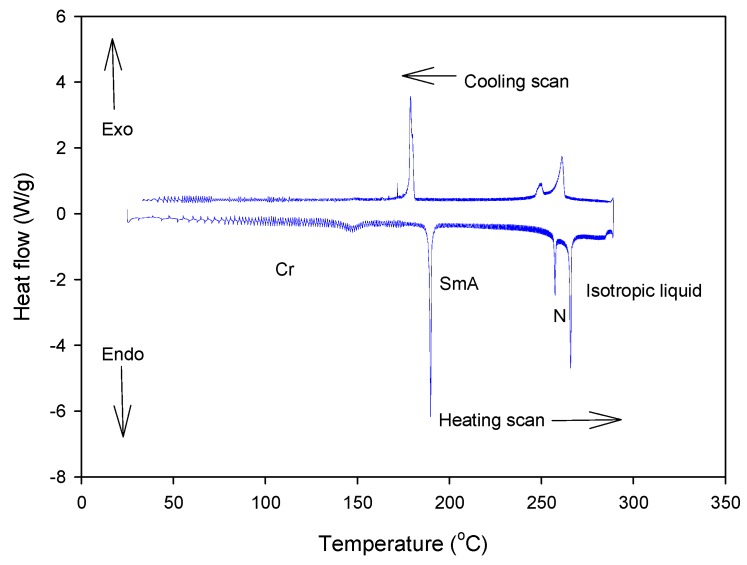
Differential scanning calorimetry (DSC) thermograms of supramolecular acid **1** upon heating and cooling scans with heating rate 10 °C/min.

Firstly, the mesomorphic behavior of the synthesized 4-[(4-(hexyloxy)phenylimino)methyl]benzoic acid dimer (**1**) was investigated by DSC and POM. DSC transitions and mesophase types were achieved and confirmed by observed textures under POM. Figure 1 illustrates the DSC thermogram upon heating/cooling scan for the prepared supramolecular acid (**1**).


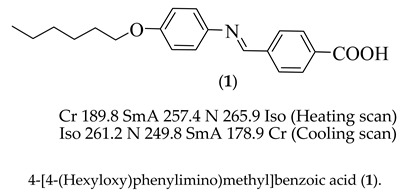


**Figure 2:**
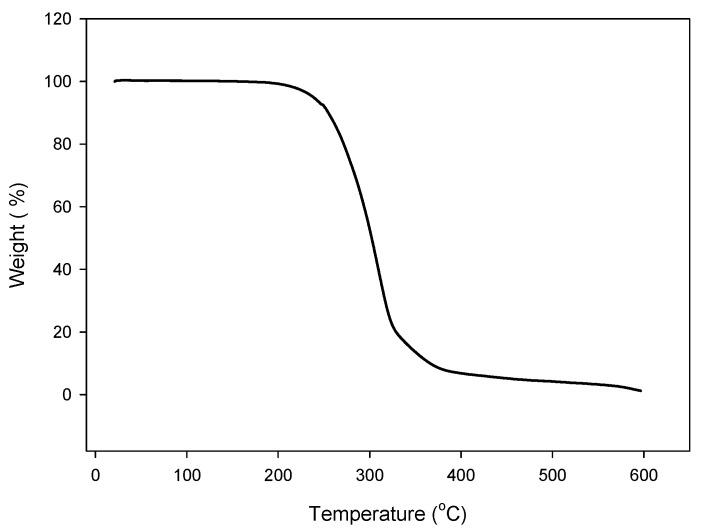
TheThermogravemetric analysis (TGA) curve of acid dimer **1** recorded under nitrogen, with a heating rate of 10 °C/min.

The phase behavior investigation revealed that the hexyloxy phenyiminobenzoic acid (**1**) is dimorphic, exhibiting an enantiotropic smectic A phase followed by a nematic phase. In Figure 1 illustrates the prepared acid (**1**) exhibits broad SmA mesophase stability (67.6 °C) and a narrow N phase stability (8.5 °C) upon heating, where it melts at 189.8 °C and changes to an isotropic liquid at ≈266.0 °C. On the other hand, the cooling of the isotropic melts showed a nematic phase at 261.2 °C and an SmA phase at 249.8 °C. Then, recrystallization was observed at 178.9 °C. The transition temperature of **1** was in agreement with that of the previous investigation [25,26]. The thermal stability of compound **1** was also confirmed by TG analysis. Figure 2 shows the TGA curve of the supramolecular acid dimer. As seen in Figure 2, thermal degradation occurs at only one step, with a maximum rate loss (T_max_) at ca. 329 °C. This indicates high thermal stability for the prepared compounds above isotropic temperatures.

**Figure 3:**
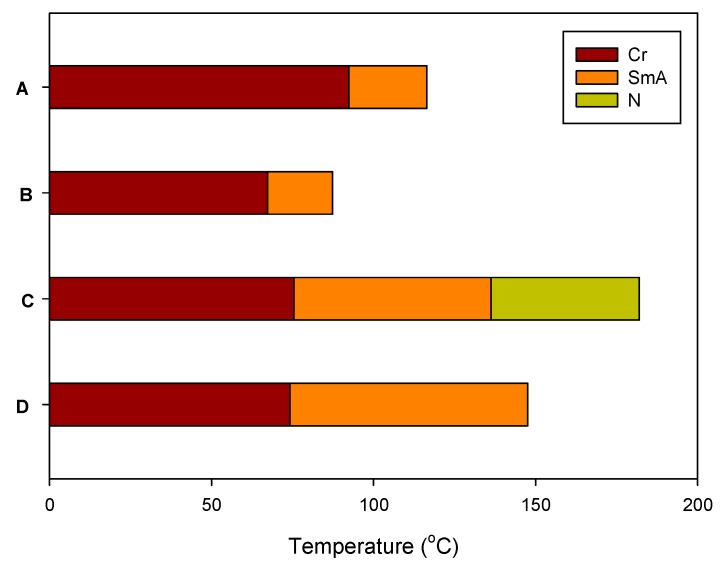
DSC transitions of series **A**–**D** upon heating.

The transition temperatures, enthalpy, and normalized entropy change of transitions as analyzed by DSC, and the identified mesophases confirmed by POM for all synthesized compounds, **A**–**D**, are summarized in Table 1. Textures and mesophase transition temperatures were achieved using the polarized optical microscope, and the results were verified by DSC measurements. Figure **3** represents graphical transition temperatures obtained from DSC thermograms, upon heating, for compounds **A**–**D**. Table 1 and Figure 3 show that all prepared compounds are enantiotropic mesomorphic with wide stability. The present mesomorphic compounds, **A**–**D**, are purely smectogenic, exhibiting a monomorphic smectic A (SmA) phase. However, compound **C** (X = I) is dimorphic and exhibits SmA and N phases. The electron-donating compound, **A**, the CH(CH_3_)_2_ substituent, exhibits an SmA phase with a thermal stability range of 24.1 °C, while the unsubstituted derivative **B** has a smectic phase with a range of 20.1 °C and a low melting temperature of 67.3 °C. It was reported in [13] that the dipole moment of the mesomeric portion of the molecule impacts the type and stability of the mesophase produced, which is dependent on the attached terminal polar substituent and the steric one that varies according to size. The halogen substituent at the terminal position showed strong influence on the mesomorphic behavior of the Schiff base molecules [30]. In our present study, the halogenated compound **C** (X = I) is dimorphic, with the highest thermal nematic stability (182.0 °C) and SmA stability (136.3 °C). The transition melting temperature of **C** is 75.4 °C, and its smectic and nematic phases’ ranges are 60.9 and 45.7 °C, respectively. While the fluoro substituted derivative **D** is monomorphic smectogenic with a wide SmA range (73.4 °C), which is lower than the range of **C** in thermal stability. This result may be attributed to the volume as well as the electronic nature of the F-atom that completely differs from the iodo atom. Therefore, the former is less easily polarized due to the electrons on this atom, which are tightly held and located closer to the nucleus [31]. Thus, the lower polarizability and intermolecular attractive force of the **D** molecule is due to the smaller size of the F-atom, which affected mesophase stability. In general, the stability of the mesophase is influenced by an increase in the polarizability and/or polarity of the mesogenic core of the molecule. This was found to be true, where in synthesized series **A**–**D**, the electron-donating (CH(CH_3_)_2_) and electron-withdrawing halogens (I and F) groups enhance the mesophase stability more than their unsubstituted derivative (**B**). 

Generally, among calamitic mesogens, there is a direct relation between mesophase behavior and the intermolecular interaction, which depends mainly upon the geometry/shape of the molecules, the polarizability anisotropy/size of the terminal polar group X, and the stereo electronic properties of the whole molecule. Therefore, in our present work, the molecular association of the rod-like molecules and, consequently, their mesophase stability (***T***c) depends mainly on the intermolecular interactions of linear molecules that differ according to the polarity and the size of the polar substituent X. The higher molecular polarizability contributed by CH(CH_3_)_2_ (I and F substituents)stabilizes the mesophase and consequently led to higher ***T***c values. The terminal substituent (X) in the molecules can be ordered according to their ability to enhance the mesophase’s range and stability: I ˃ F ˃ CH(CH_3_)_2_ ˃ H. 

**Figure 4:**
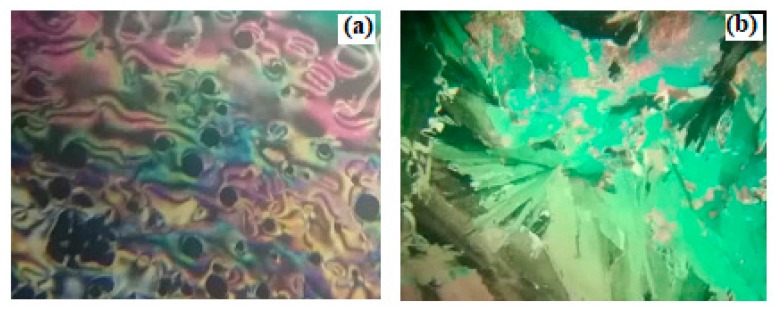
Polarized optical microscopy (POM) textures upon heating for (**a**) the nematic phase of compound **1** at 262.0 °C and (**b**) the smectic A phase of compound **D** at 120.0 °C.

The competitive interaction between end-to-end intermolecular aggregation and side-side parallel interaction plays an important role in determining the type and stability of the enhanced mesophase, as well as their transitions to anisotropic phase and the development of the smectic phase, established by the predominance of lateral attractions. The types of the observed mesophase textures were identified for the prepared compounds, and two examples are represented in Figure 4.

### 3.3. Comparable Study of Three Schiff base/ester Positional Isomers

The investigated phenyl 4-[(4-(hexyloxy)phenylimino)methyl]benzoate (**B**) is compared with that of previously investigated Schiff base/ester derivatives,4-phenyliminomethyl)phenyl 4’−hexyloxybenzoate (**E**), [24]and 4-(benzylideneamino)phenyl 4’-hexyloxybenzoate (**F**) [16]. It was found that from the comparison between their mesophase stabilities, the compound **E** is relatively higher in thermal stability than those of its isomers **B** and **F**. Isomer **E** is different than the prepared isomer **B** in the location and the inversion of the ester linkage, as well as in the exchange in the position of terminal substituents. Thus, it seems that the attachment of a hexyloxy chain into the terminal ring, which attached to the ester group rather than the (-CH=N-) mesogen, increases the polarizability and, consequently, enhances the terminal intermolecular association between molecules, showing a nematic phase. The results confirmed that the effect of the alkoxy chain attached to the ester linkage is more effective on the type and stability of phases when compared with the chainsattached to the azomethine moiety.

### 3.4. DFT Calculations

#### 3.4.1. The Geometrical Structure

The geometrical structure and other properties (the energy gap between the frontier molecular orbitals, the charge distribution, polarizability, λ_max_ of UV-Vis absorption spectra, dipole moment, and the mesophase stability of the liquid crystalline) of organic compounds could be highly affected by the electronic nature and position of the attached substituents. It is well known that the Hammett substituent coefficient (σ) and the inductive sigma constant (I) are major parameters that could be used to evaluate the electronic effect of the substituents upon the reaction rate, as well as the reaction mechanism [32]. Consequently, it is worthy to investigate the relationship between the σ-coefficient and the I-constant with variable parameters that affect the characteristics of the liquid crystalline materials. 

**Figure 5:**
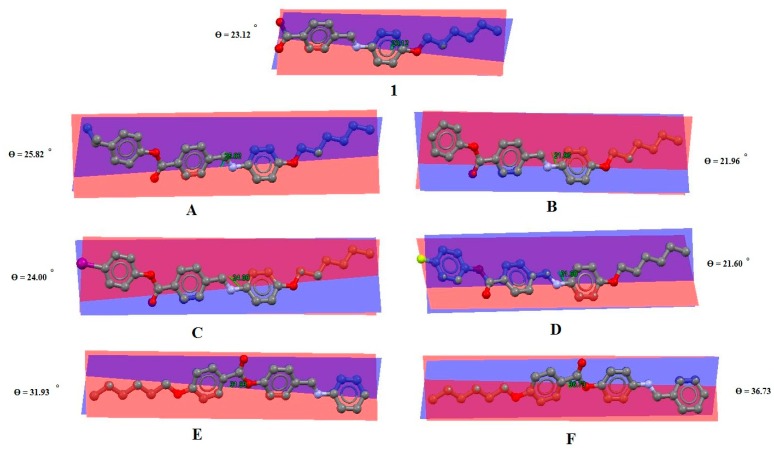
Estimated geometrical structure and twist angles of compounds **1** and **A**–**F**.

The optimum geometrical structure of all investigated compounds **1** and **A–D** were calculated in the gas phase using method DFT/B3LYP for the base set LANL2DZ. The results of the DFT calculations revealed that all compounds are in non-planar geometries, and the comparative studies between the compounds under investigation with the previously prepared compounds with different positions and orientations of the mesogenic cores **E** [24] and **F** [16]have been illustrated. Obviously, the deviation from planarity depends upon the nature of the attached substituent and the position of the mesogenic cores (COO, CH=N) of the liquid crystalline compounds. The twist angle ϴ between the two phenyl rings attached by the C=N linking group is affected by the electronic nature of the terminal substituent X (Figure 5, Table 2).

As shown in Figure 5, the twist angle ϴ between the two phenyl rings attached with the C=N group of the free acid **1** is ϴ = 23.12°. However, a small decrease to 21.96° is caused by the esterification of the acid with an unsubstituted phenol. The attachment of the electron donating group decreases the planarity by 2.6° with respect to the free acid. The high electron drawing the F-atom increases the planarity to ϴ = 21.60°. However, the presence of the iodo-atom in the para position increases the twist angle ϴ to 24.00°.These results illustrate that the twist angle ϴ is impacted by the resonance effect of the substituents, rather than their inductive effects. Moreover, changing the position and the orientation of the mesogenic cores (C=N and COO) affects the twist angle largely to be ϴ = 31.93° for **E** and 36.73° for its positional isomer, **F**.

The electronic nature of terminal X has a significant effect on the planarity of the investigated compounds **A**–**D**. On the other hand, the size of the terminal substituent X does not impact the degree of the deviation from planarity where this substituent is separated from these systems (C=N) by another benzene. Moreover, the orientation of the C=N of the isomers **E** and **F** of the unsubstituted derivative **B** has a pronounced effect on the planarity of these isomers.

**Figure 6:**
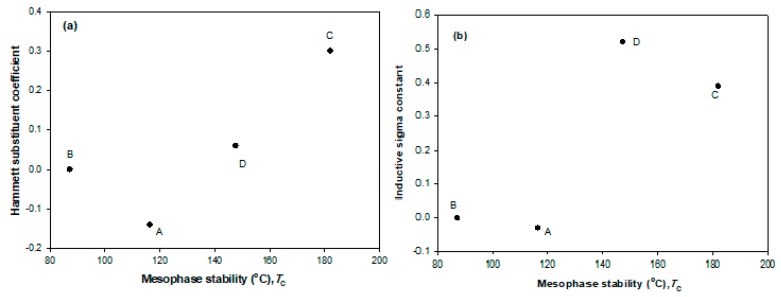
Dependence of the ***T***c against (**a**) Hammett substituent coefficient (σ), (**b**) inductive sigma constant (σI) of compounds **A**–**D**.

**Figure 7:**
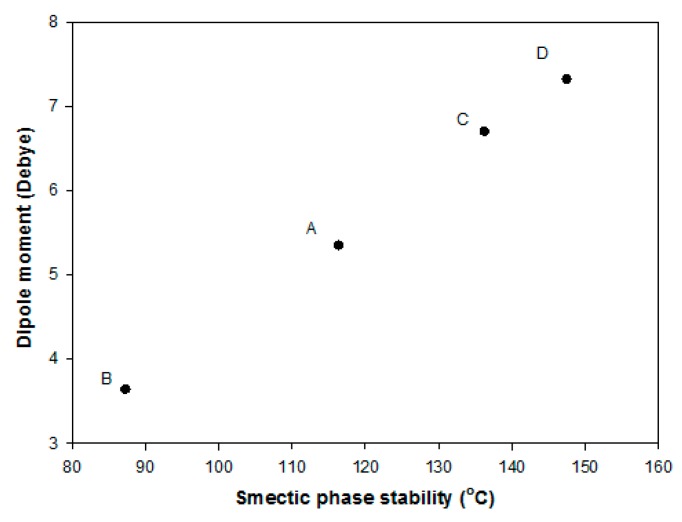
Dependence of the ***T***_SmA_ against the dipole moment µ of compounds **A**–**D**.

The calculated thermal parameters, dipole moment, and polarizability of the investigated liquid crystalline materials **1** and **A**–**F** are tabulated in Table 3. As shown in Figure 6, the dipole moment is highly impacted by the electronic nature of the substituent X, as well as the position and the orientation of the mesogenic core. The largest electron withdrawing group (F-atom) showed the highest dipole moment (µ = 7.3 Debye). The results of the calculated dipole moment illustrate the type of the mesomorphic properties of the investigated compounds. The terminally neat derivative, **B**, with the lowest polarity (µ = 3.6 Debye) and van der Waal’s volume (3.5 cm^3^/mol), makes the smectic mesophase range the narrowest (20 °C). However, the large van der Waal volume of the isopropyl group of derivative **A** (44.34 cm^3^/mol) is expected to increase the smectic mesophase’s stability due to a higher degree of aggregation interaction. On the other hand, the high dipole moment of the largest inductive sigma constant F-atom (0.52) makes a significant lateral interaction that enhances smectic mesophase with the highest range of the smectic mesophases (73.4 °C). On the other hand, the iodo derivative (**C**), with a large van der Waal’s volume (19.34 cm^3^/mol), the highest Hammett substituent constant (0.30), and the least planarity, decreases the packing and decrements the smectic mesophase stability to 136 °C instead of 147 °C for the fluoro derivative. This enhances end to end interactions to give the nematic mesophase a very wide range (up to 45.4 °C). Obviously, the position and the orientation of the mesogenic core have a high impact on the estimated thermal parameters as well as the experimental mesomorphic properties. The low value of the dipole moment of the isomers **E** and **F** and the high degree of non-planarity do not allow a close packing with a high parallel interaction to enhances the smectic mesophase, leaving the terminal interaction to predominantly show a nematic mesophase. Moreover, neither the dipole moment nor the polarizability have been impacted by the orientation C=N group. The differences in the nematic mesophase stability between the two isomers could be attributed to their geometry. The lower planarity of compound **F** lowers the mesophase stability compared to its more planar isomer (**E**) (***T***c = 149.0 and 156.8 °C, respectively (Figure 6 and Figure 7)).

**Figure 8:**
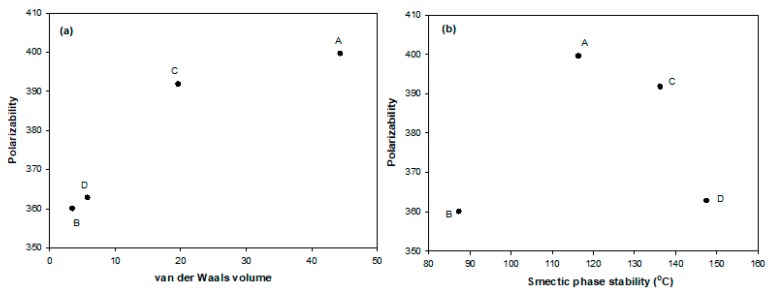
Correlation between the polarizability against (**a**) the van der Waal volume of terminal X and (**b**) the smectic mesophase stability ***T***_SmA._

Polarizability [33,34,35] could be considered another factor affecting the type as well as the phase stability temperature, ***T***c. To illustrate the impact of these factors, the polarizability of substituent X is displayed graphically against the van der Waal volume of the substituent (Figure 8a) and the ***T***_SmA_ values (Figure 8b). The results suggest that the van der Waal volume impacts the polarizability due to the attachment of the substituent, X; as the volume increases, the polarizability increases. As seen in Figure 8b**,** the mesophase stability ***T***_SmA_ of the compounds under investigation is obviously affected by polarizability, except for the unsubstituted derivative (**B**). However, this result occurs not only because polarizability is the main factor affecting the mesophase stability but also because of the polar nature and van der Waal’s volume, as well as the close packing ability due to the geometrical effects enhancing the intermolecular attraction between molecules and bulk groups that facilitate molecular space-filling [36].

#### 3.4.2. Optical Properties and Frontier Molecular Orbitals 

**Figure 9:**
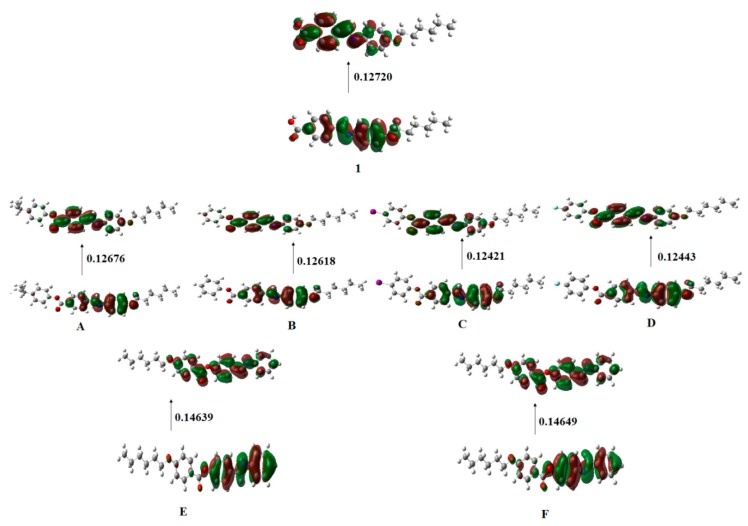
The calculated ground state isodensity surface plots for frontier molecular orbitals of compounds **1** and **A**–**F**.

**Figure 10:**
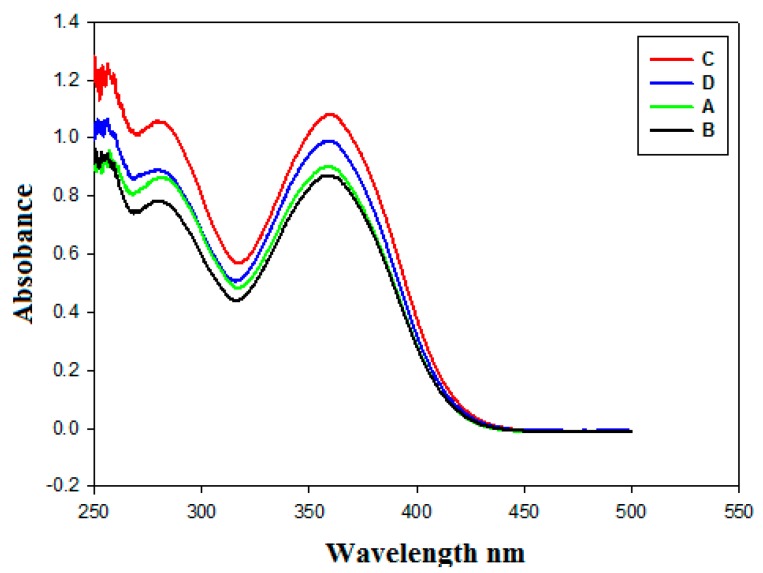
UV–vis spectra of compounds **A**–**D** in dichloromethane at 25 °C.

**Figure 11:**
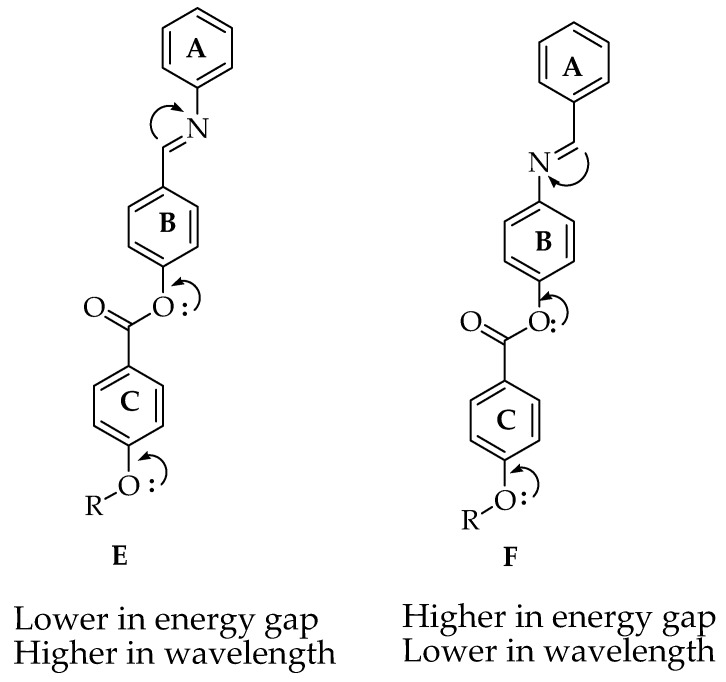
Resonance effect of compounds **E, F**.

Optical applications of non-linear optical (NLO) liquid crystals are highly affected by the energy difference between the frontier molecular orbitals (FMOs), HOMO (highest occupied molecular orbital), and LUMO (lowest unoccupied molecular orbital) [37,38]. Moreover, the energy gap between FMOs is a good tool for the prediction of important parameters, such as chemical hardness (**η**), global softness (**S**), and polarizability (**α**). Figure 9 illustrates the calculated ground state isodensity surface plots for the FMOs of compounds **1** and **A**–**F**. Table 4 summarizes the values of the FMO energy gap and the global softness (**S**) and experimental λ_max_. As shown in Table 4 and Figure 9 and Figure 10, the global softness and the FMO energy gaps, as well as the experimental maximum absorption (λ_max_), have not been significantly affected by van der Waal’s volume or the electronic nature of the terminal substituent. This can be explained by the fact that the substituents do not participate in HOMO and LUMO orbitals. The experimental results of the UV-Vis absorption are constituent with those of the theoretical calculations of the energy gap between the FMO. However, the energy gap between the FMO is highly affected by the orientation and position of the mesogenic core (COO and C=N). The λ_max_ decreases by about 60 nm by changing the position of the mesogenic cores of the unsubstituted derivative (**B**) compared with its isomers, **E** and **F**. However, the orientation of the C=N group of **E** and **F** has little effect on either the ΔE of the FMOs or the experimental UV-Vis absorption, only 15 cm^−1^ of Δλ_max_ and 0.001a.u for ΔE. Moreover, as can be concluded from Table 4**,** the terminally neat derivative (**B**) is softer than both its positional and orientational isomers (**E** and **F**). The orientation of C=N of compound **E** is more proper than **F** for resonance. This orientation cloud permits the maximum delocalization of the π-electrons and, consequently, decreases the ΔE of the frontier’s molecular orbitals, Figure 11.

#### 3.4.3. Molecular Electrostatic Potential (MEP)

**Figure 12:**
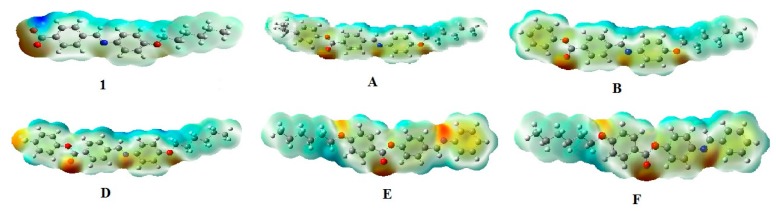
Electrostatic potentials (MEP) of the prepared compounds.

The charge distribution map for the compounds **1** and **A**–**F** was calculated using the same basis sets according to molecular electrostatic potential (MEP, Figure 12). The red region (negatively charged atomic sites) was localized on the oxygen atoms and the nitrogen atom of the imino group, while alkyl chains showed the least negatively charged atomic sites (blue regions). As shown in Figure 12, the position and orientation of the core, as well as the electronic nature of the terminal substituent (X), affect the distribution of the charge map. This could impact the type and stability of the mesophase by altering the competitive interaction between end-to-end and side-side interactions. Recently, we reported the relationship between the theoretical charge distribution and experimental mesophase type [18,39,40]. The alteration of the charge distribution on the molecules due to a greater electron donation or electron acceptance could predominate terminal aggregations to enhance the nematic mesophase or the parallel interactions to give a semectic mesophase. 

## 4. Conclusions

Schiff base liquid crystals of a Schiff base/ester series with a terminal polar substituent were successfully synthesized and thermally characterized. Molecular structures were confirmed via elemental analyses, FT-IR, and NMR spectroscopy. The thermal, mesomorphic, and optical properties of the newly prepared compounds were investigated by TGA, DSC, POM, and UV- spectroscopy.

The study revealed that:

The Schiff base supramolecular acid is enantiotropic dimorphic with high thermal stability in the SmA and N mesophases. All synthesized Schiff base/ester compounds are mesomorphic, exhibiting a wide SmA range of high thermal stability. However, the iodo derivative is dimorphic with the SmA and N phases. The mesomorphic properties are greatly impacted by the size and polarity of the terminal substituents, and the unsubstituted derivative shows the smallest range of SmA mesophase stability. The positional and orientational inversion of the mesogenic cores (COO, C=N) have a pronounced effect on the type and the stability of the observed mesophase. DFT and theoretical calculations of geometrical parameters revealed that the twist angle ϴ between the two phenyl rings attached with a C=N linkage is impacted by the resonance effect of the terminal substituents and plays an important role in enhancing the mesophase transition stability and its range.

## Figures and Tables

**Table 1:** Phase transition temperatures (°C), enthalpy of transitions (kJ/mol), and normalized transition entropy temperature for compounds **A**–**D**.

Compound	X	*T*_Cr-SmA_	*∆H*_Cr-SmA_	*T*_SmA-I_	*∆H*_SmA-I_	*∆S*_SmA-I_/R	*T*_N-I_	*∆H*_N-I_	*∆S*_N-I_/R
**A**	CH(CH_3_)_2_	92.3	28.76	116.4	2.33	2.41	-	-	-
**B**	H	67.3	28.38	87.3	1.92	2.65	-	-	-
**C**	I	75.4	26.87	136.3	3.11	2.74	182.0	1.87	1.24
**D**	F	74.1	25.48	147.5	1.80	1.47	-	-	-

Abbreviations: ***T***_Cr-SmA_ = crystal to smectic A transition; ***T***_SmA-I_ = Smectic A to isotropic liquid transition; ***T***_N-I_ = Nematic to isotropic liquid transition. ***∆H***_Cr-SmA_ = crystal to smectic A transition; ***∆H***_SmA-I_ = Smectic A to isotropic liquid transition; ***∆H***_N-I_ = Nematic to isotropic liquid transition. ***∆S***_SmA-I_/R _=_Smectic A to isotropic liquid transition; ***∆S***_N-I_/R = Nematic to isotropic liquid transition.

**Table 2:** Hammett substituent coefficient (σ) and the van der Waal volume of terminal X for compounds **A**–**D**.

Compound	van der Waal Volume	Hammett Substituent Coefficient (σ)	Inductive Sigma Constant (I)
**A**	44.34	−0.14	−0.03
**B**	3.50	0.00	0.00
**C**	19.64	0.30	0.39
**D**	5.80	0.06	0.52

**Table 3:** Thermal parameters (Hartree/Particle), Dipole Moment (Debye), and polarizability of compounds **1** and **A**–**F**.

Parameter	1	A	B	C	D	E	F
**E_corr_**	0.392807	0.559305	0.474386	0.463692	0.465729	0.474707	0.474454
**ZPVE**	−1055.849580	−1404.623757	−1286.783451	−1297.569307	−1386.035695	−1286.786475	−1286.785911
**E*_tot_***	−1055.825993	−1404.591179	−1286.755255	−1297.539432	−1386.006571	−1286.758403	−1286.757712
**H**	−1055.825049	−1404.590235	−1286.754311	−1297.538488	−1386.005627	−1286.757458	−1286.756768
**G**	−1055.907119	−1404.694419	−1286.849136	−1297.638567	−1386.102863	−1286.850666	−1286.851627
**Dipole moment**	9.4330	5.3484	3.6413	6.6999	7.3187	3.3748	3.2828
**Polarizability α**	279.08	399.61	360.04	391.80	362.78	347.25	346.93

ZPVE: sum of electronic and zero-point energies; E_tot_: sum of electronic and thermal energies; H:sum of electronic and thermal enthalpies; G:sum of electronic and thermal free energies.

**Table 4:** Molecular orbital energies, hardness (**η**), and global softness (**S**) of compounds **A**–**F**.

Compound	E_HOMO_ (a.u)	E_LUMO_ (a.u)	ΔE(E_LUMO_-E_HOMO_) (a.u)	S= 1/ΔE	λ_maxexp_
**1**	−0.22052	−0.09332	0.12720	7.861635	358.6
**A**	−0.21376	−0.08700	0.12676	7.888924	358.9
**B**	−0.21486	−0.08868	0.12618	7.925186	358.8
**C**	−0.21706	−0.09285	0.12421	8.050882	360.4
**D**	−0.21742	−0.09299	0.12443	8.036647	359.4
**E**	−0.21773	−0.07134	0.14639	6.831068	278.0
**F**	−0.21979	−0.07330	0.14649	6.826405	263.0

## Supplementary Materials

The following are available online. Figure S1: ^1^H NMR of 4-[(4-(hexyloxy)phenylimino)methyl]benzoic acid (1), Figure S2: C^13^ NMR of 4-[(4-(hexyloxy)phenylimino)methyl]benzoic acid (1), Figure S3: ^1^H NMR of phenyl 4-[(4-(hexyloxy)phenylimino)methyl]benzoate (B), Figure S4: ^1^H NMR of 4-iodophenyl 4-[(4-(hexyloxy)phenylimino)methyl]benzoate (C), Figure S5: ^1^H NMR of 4-iodophenyl 4-[(4-(hexyloxy)phenylimino)methyl]benzoate (C), Figure S6: ^1^H NMR of 4-fluorophenyl 4-[(4-(hexyloxy)phenylimino)methyl]benzoate (D), Figure S7: ^1^H NMR of 4-fluorophenyl 4-[(4-(hexyloxy)phenylimino)methyl]benzoate (D), Figure S8: FTIR of 4-isopropylphenyl 4-[(4-(hexyloxy)phenylimino)methyl]benzoate (A), Figure S9: FTIR of phenyl 4-[(4-(hexyloxy)phenylimino)methyl]benzoate (B), Figure S10: FTIR of 4-iodophenyl 4-[(4-(hexyloxy)phenylimino)methyl]benzoate (C), Figure S11: FTIR of 4-fluorophenyl 4-[(4-(hexyloxy)phenylimino)methyl]benzoate (D), Figure S12: DSC thermograms of compound B upon heating and cooling cycles at heating rate 10 °C/min.
