# Supplementary material for: The Synthesis of New Thermal Stable Schiff Base/Ester Liquid Crystals: A Computational, Mesomorphic, and Optical Study
Source: Molecules. 2019 Aug 21;24(17):3032. doi: 10.3390/molecules24173032 (PMC6749398; doi:10.3390/molecules24173032)
Supplement: Supplementary file 1 [file molecules-24-03032-s001.pdf]

Supplementary Data

Synthesis of new thermal stable Schiff base/ester liquid crystals; computational, mesomorphic and optical study

M. Hagar [1,2*], H.A. Ahmed [1,3*], Sherif S. Nafee [4,5], Reda M. El-Shishtawy [6,7], and Bahaaudin M. Raffah [4]

1. College of Sciences, Chemistry Department, Yanbu, Taibah University, Saudi Arabia., (MH) mohamedhaggar@gmail.com, (HA) ahoda@sci.cu.edu.eg.
2. Faculty of Science, Chemistry Department, Alexandria University, Alexandria, Egypt.
3. Faculty of Science, Department of Chemistry, Cairo University, Cairo, Egypt.
4. Physics Department, Faculty of Science, King Abdulaziz University, Jeddah 21589, Saudi Arabia, (SN) snafee@kau.edu.sa; (BR) BRAFFAH@kau.edu.sa
5. Physics Department, Faculty of Science, Alexandria University, Alexandria 21121, Egypt.
6. Department of Chemistry, Faculty of Science, King Abdulaziz University, Jeddah 21589, Saudi Arabia, (RE) relshishtawy@kau.edu.sa.
7. Dyeing, Printing and Textile Auxiliaries Department, Textile Research Division, National Research Center, Dokki, Cairo 12622, Egypt.

*Correspondence: M. Hagar , mohamedhaggar@gmail.com; H.A. Ahmed, ahoda@sci.cu.edu.eg.

Instrumentations

Differential Scanning Calorimeter, TA instrument Co. Q20 (DSC; USA), was used for calorimetric measurements. The melting point and enthalpy of indium and lead was used for DSC calibration. Aluminum pans and (2–3 mg) sample amounts were used for DSC measurements investigation. (30 ml/min) nitrogen gas inert atmosphere and 10°C/min heating rate were selected for all measurements and all transition were recorded from the second heating scan. The types of the mesophase texture were identified by a standard polarized optical microscope (POM, Wild, Germany) with Mettler FP82HT hot stage and the temperature controller was attached thermocouple for temperature measurements. All recorded values were made twice and the results have accuracy ± 0.2°C for transition temperature.

Thermogravemetric analysis (TGA) was carried out using Shimadzu TGA-50H Thermal Analyzer under nitrogen at 10 ^{o}C/min. The experiments were conducted from room temperature up to 600 ^{o}C, and the reference material was a-alumina. The sample weights for all the experiments were taken in the range of 3–4 mg.

The purity of the prepared compounds was checked by Thin-layer chromatography (TLC, E Merck). FT-IR (Nicolet iS 10 Thermoscientific), NMR spectra (Varian EM 350L 300 MHz spectrometer (Oxford, UK)) and elemental analyses (Thermo Scientific Flash 2000 CHS/O

Elemental Analyzer, Milan, Italy) were used to confirm the structures. NMR as well as FT-IR spectroscopy showed the presence of the expected aliphatic proton for all investigated compounds.

The spectrophotometer technique carried out by UV-1800 SHIMADZU, Japan of a wavelength ranging from 200–800 nm with normal incidence of light at 27 ^{o}C was used for all compounds.

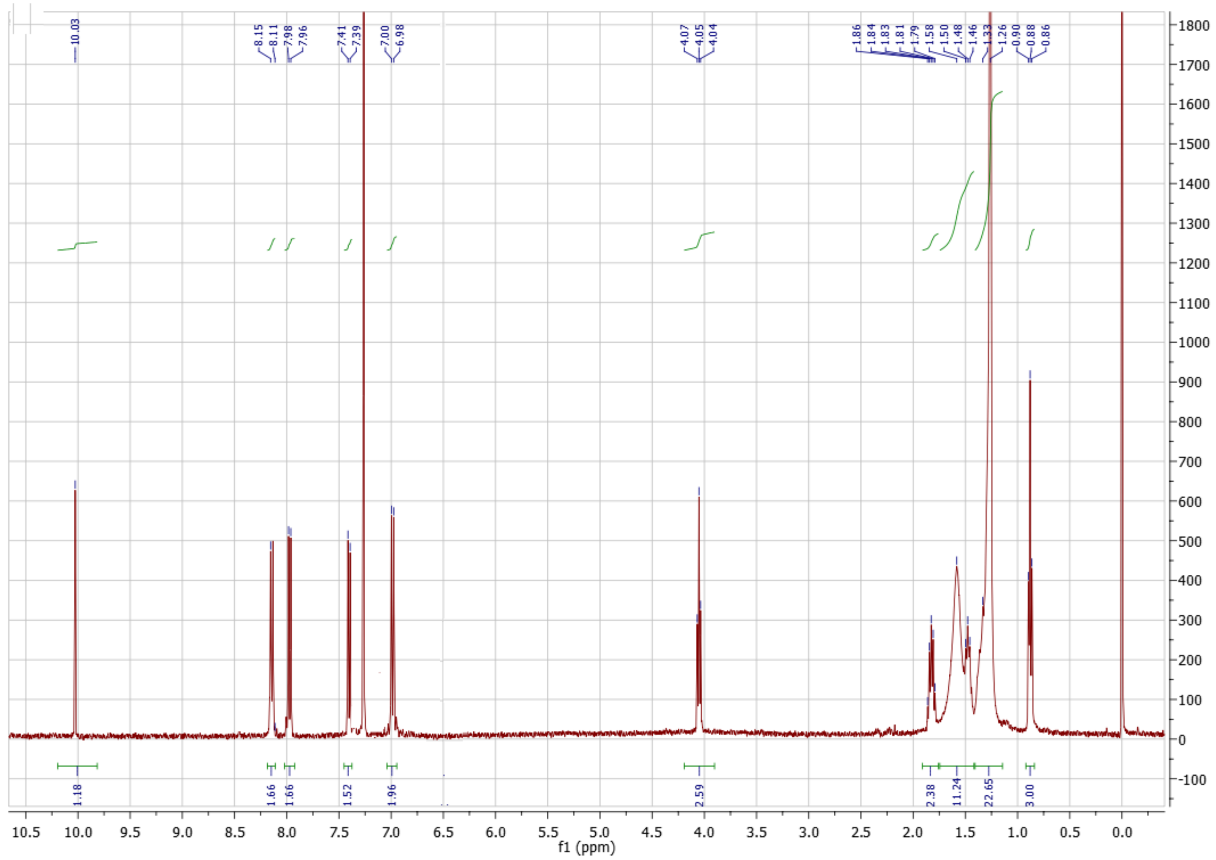

Figure S1: ^{1}H NMR of 4-[4-(hexyloxy)phenylimino)methyl]benzoic acid (1)

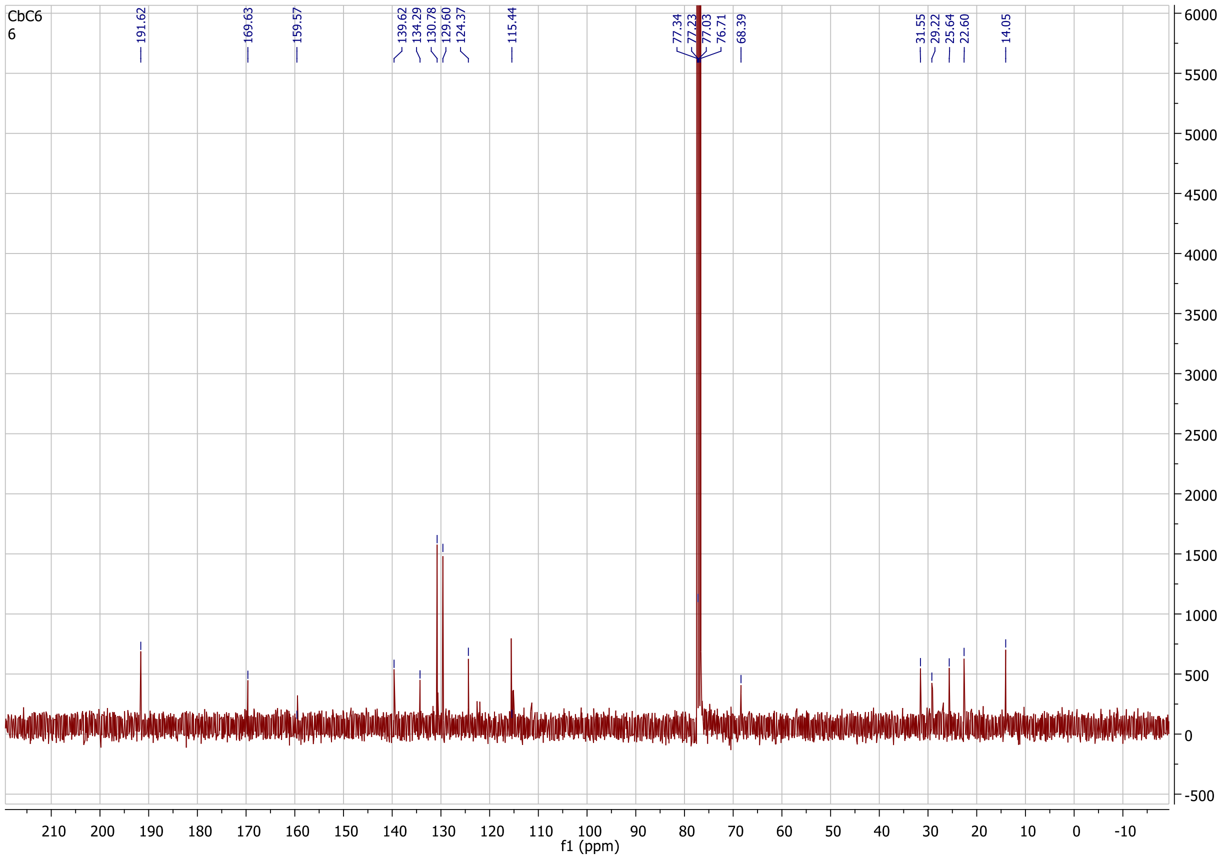

Figure S2: C^{13} NMR of 4-[4-(hexyloxy)phenylimino)methyl]benzoic acid (1)

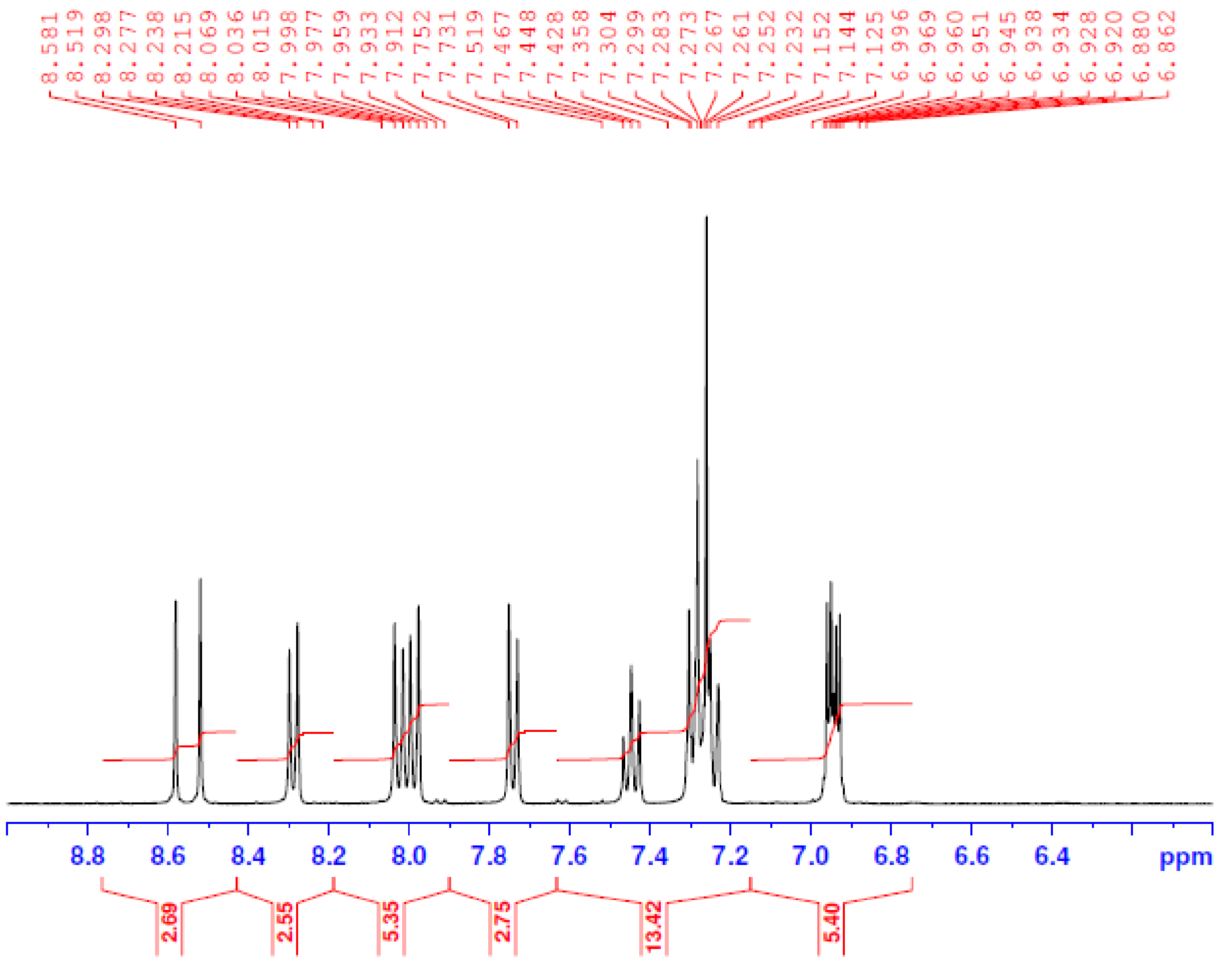

Figure S3: ^{1}H NMR of phenyl 4-[4-(hexyloxy)phenylimino)methyl]benzoate (B)

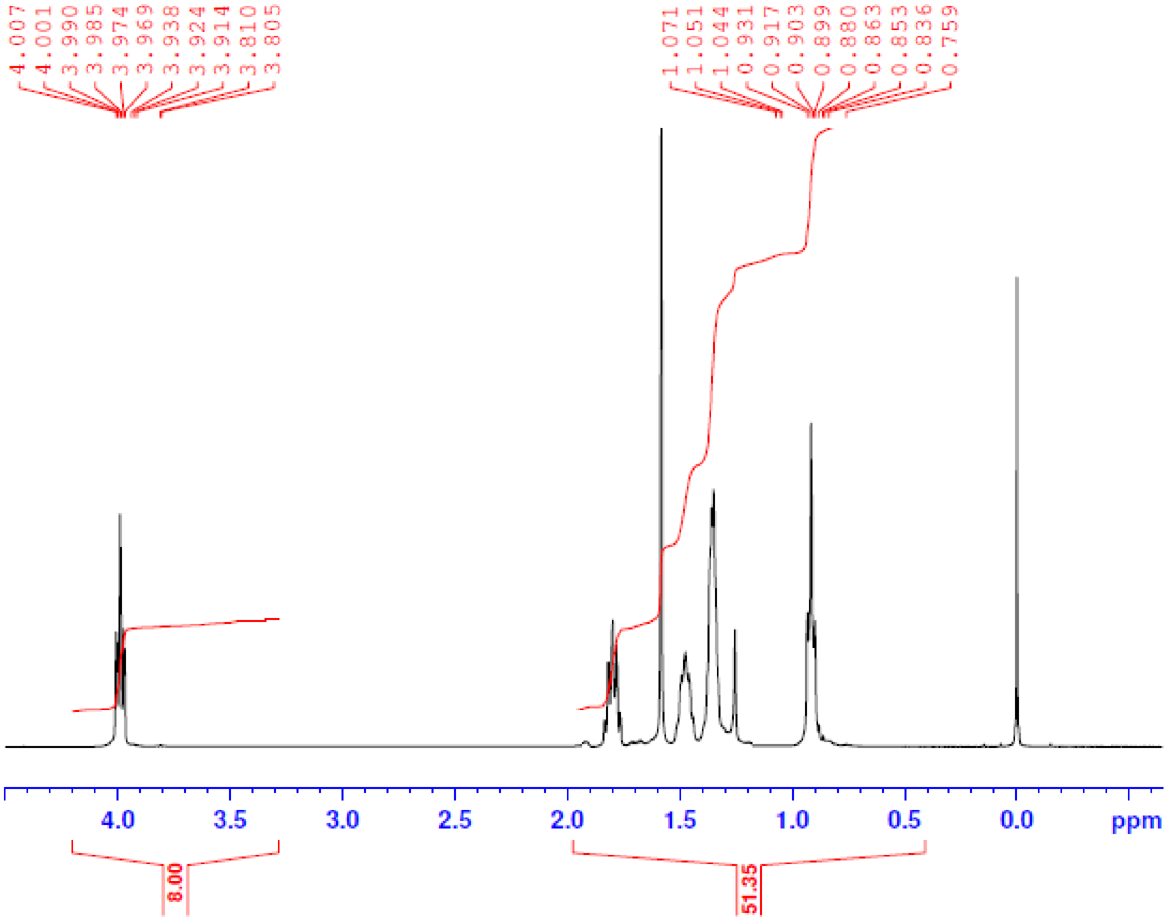

Figure S4: ^{1}H NMR of 4-iodophenyl 4-[4-(hexyloxy)phenylimino)methyl]benzoate (C)

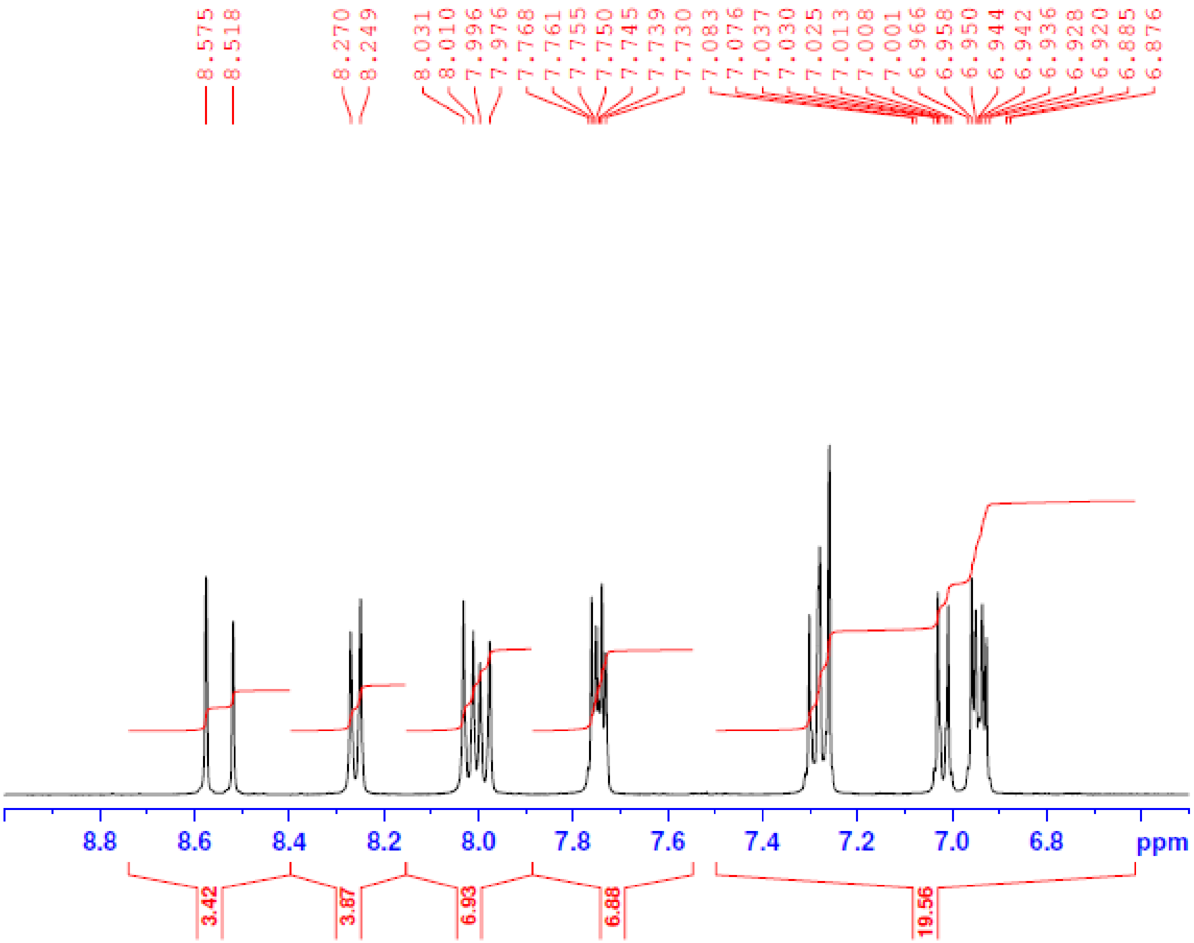

Figure S5: ^{1}H NMR of 4-iodophenyl 4-[4-(hexyloxy)phenylimino)methyl]benzoate (C)

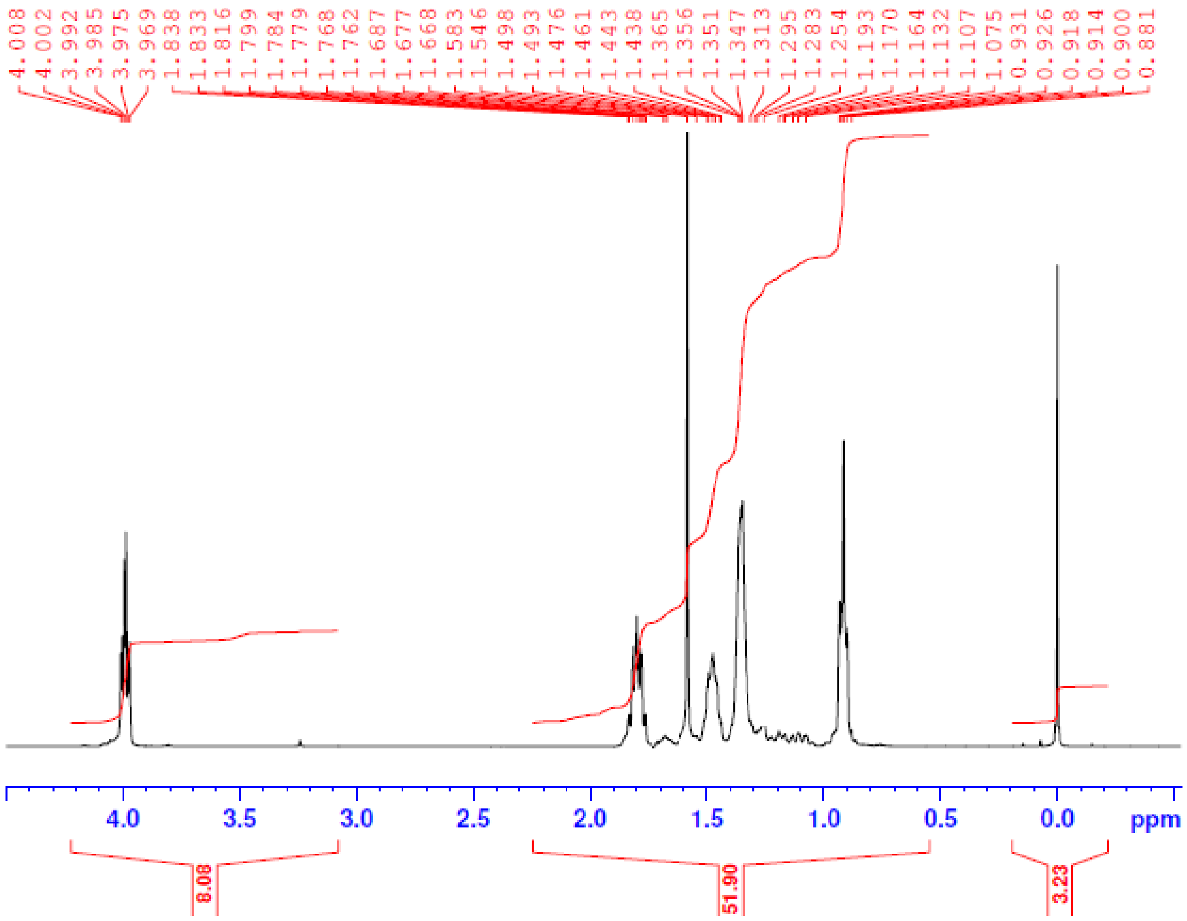

Figure S6: ^{1}H NMR of 4-fluorophenyl 4-[4-(hexyloxy)phenylimino)methyl]benzoate (D)

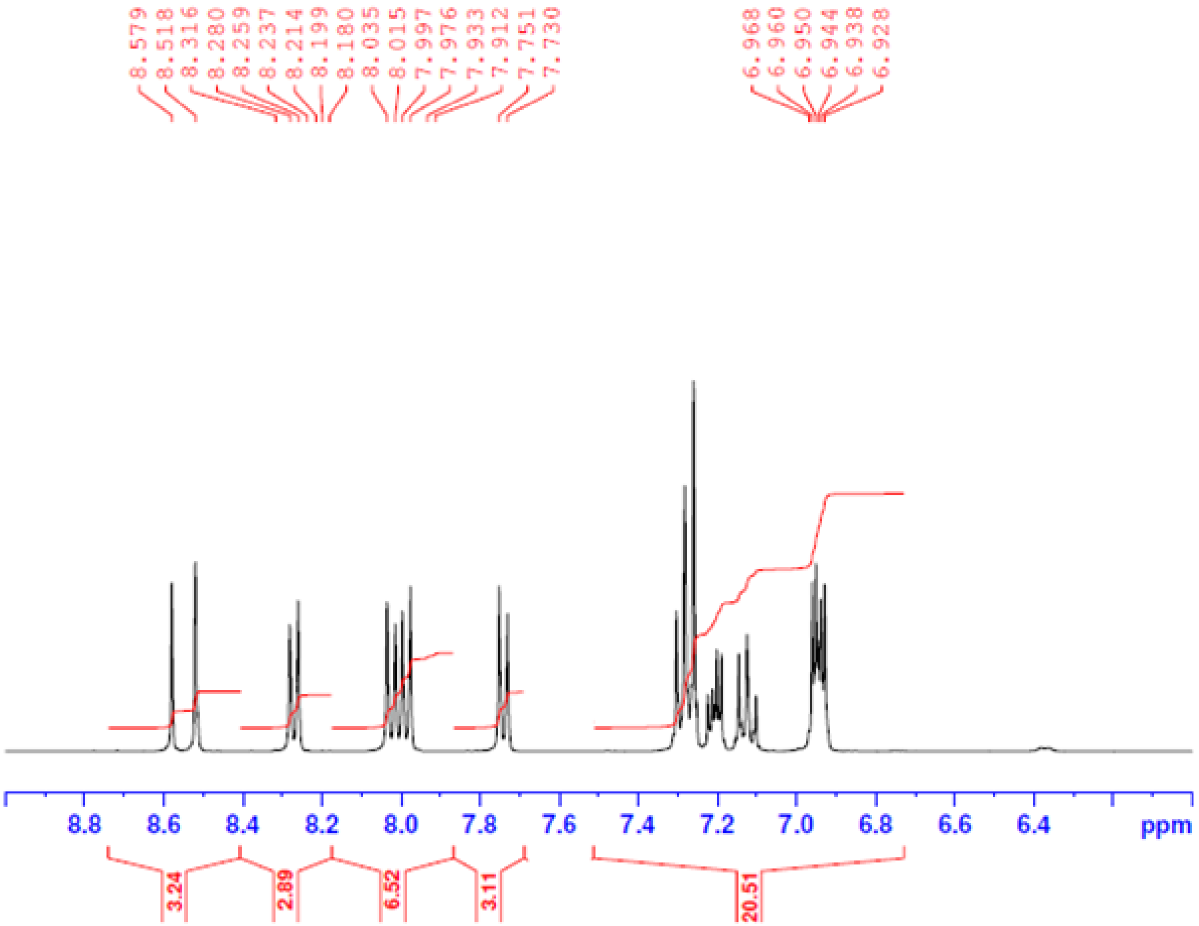

Figure S7: ^{1}H NMR of 4-fluorophenyl 4-[4-(hexyloxy)phenylimino)methyl]benzoate (D)

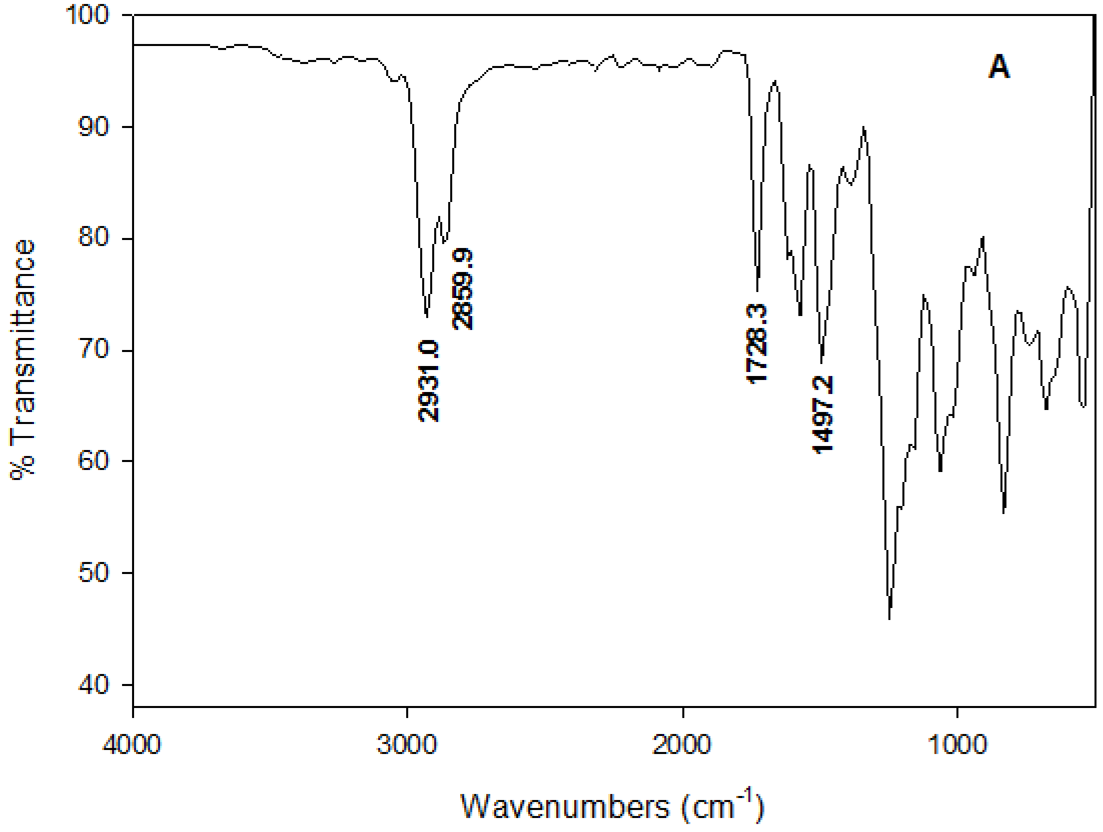

Figure S8: FTIR of 4-isopropylphenyl 4-[4-(hexyloxy)phenylimino)methyl]benzoate (**A**)

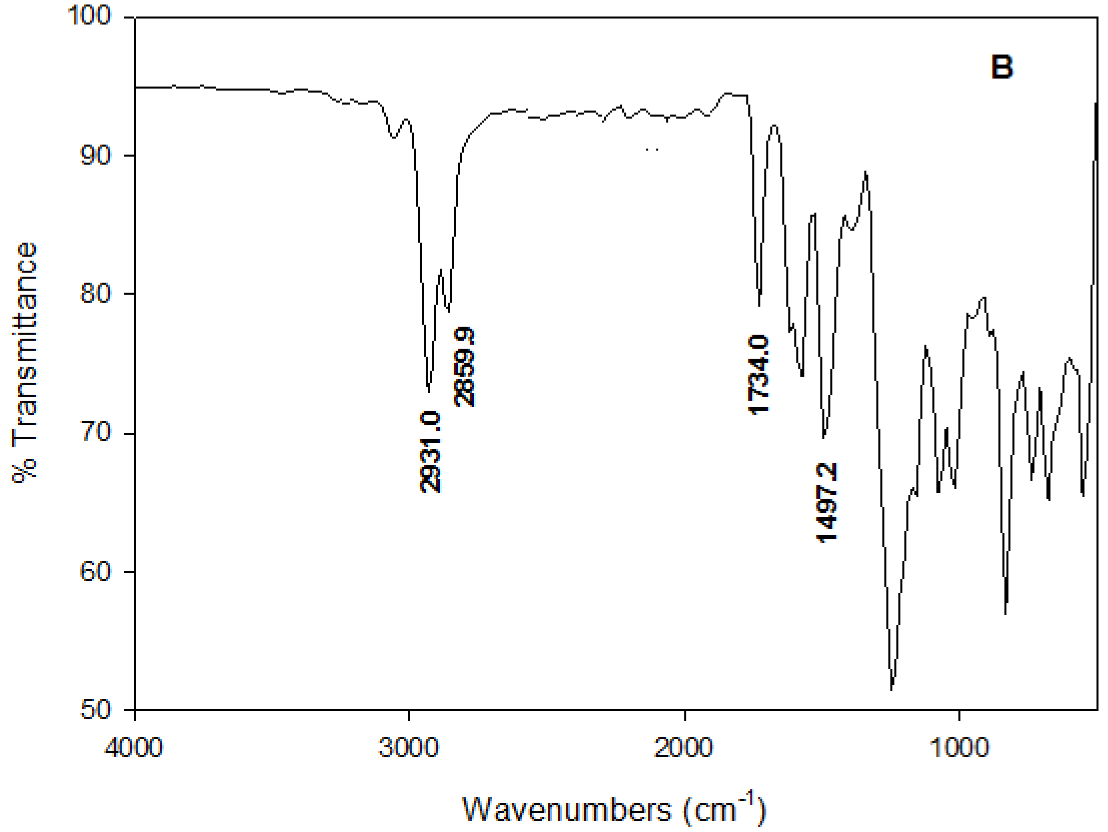

Figure S9: FTIR of phenyl 4-[4-(hexyloxy)phenylimino)methyl]benzoate (**B**)

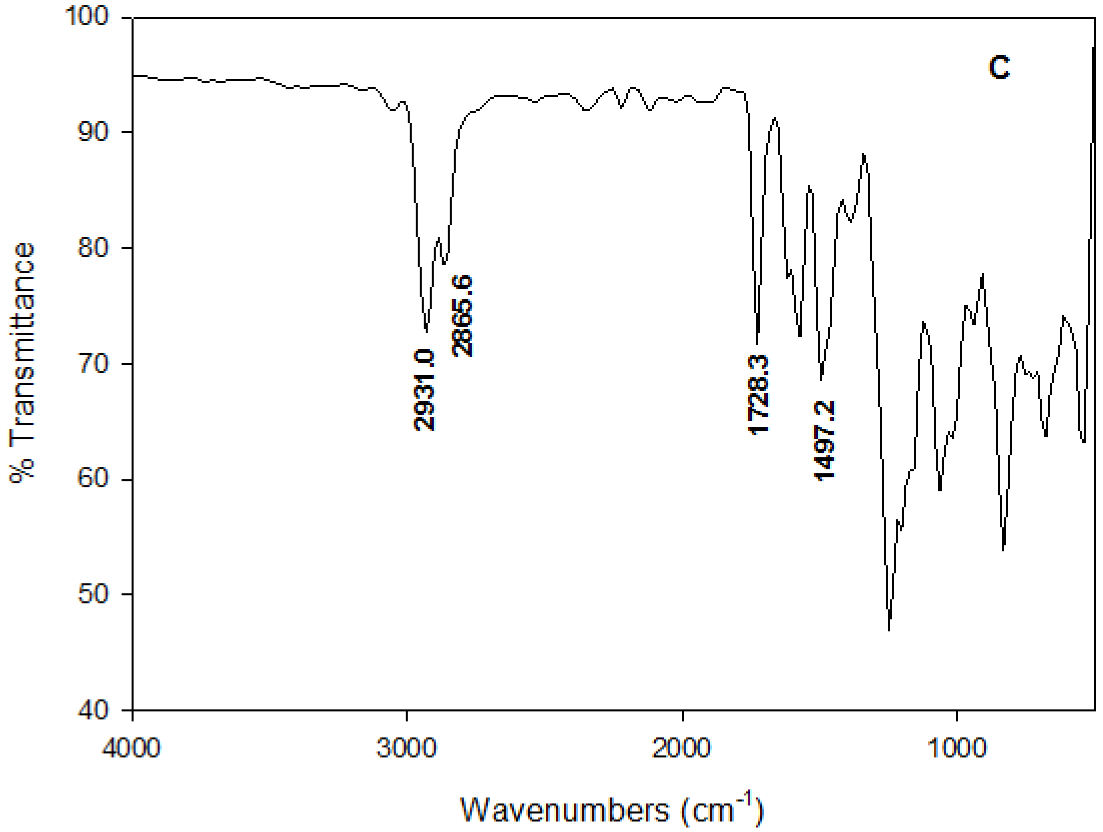

Figure S10: FTIR of 4-iodophenyl 4-[4-(hexyloxy)phenylimino)methyl]benzoate (C)

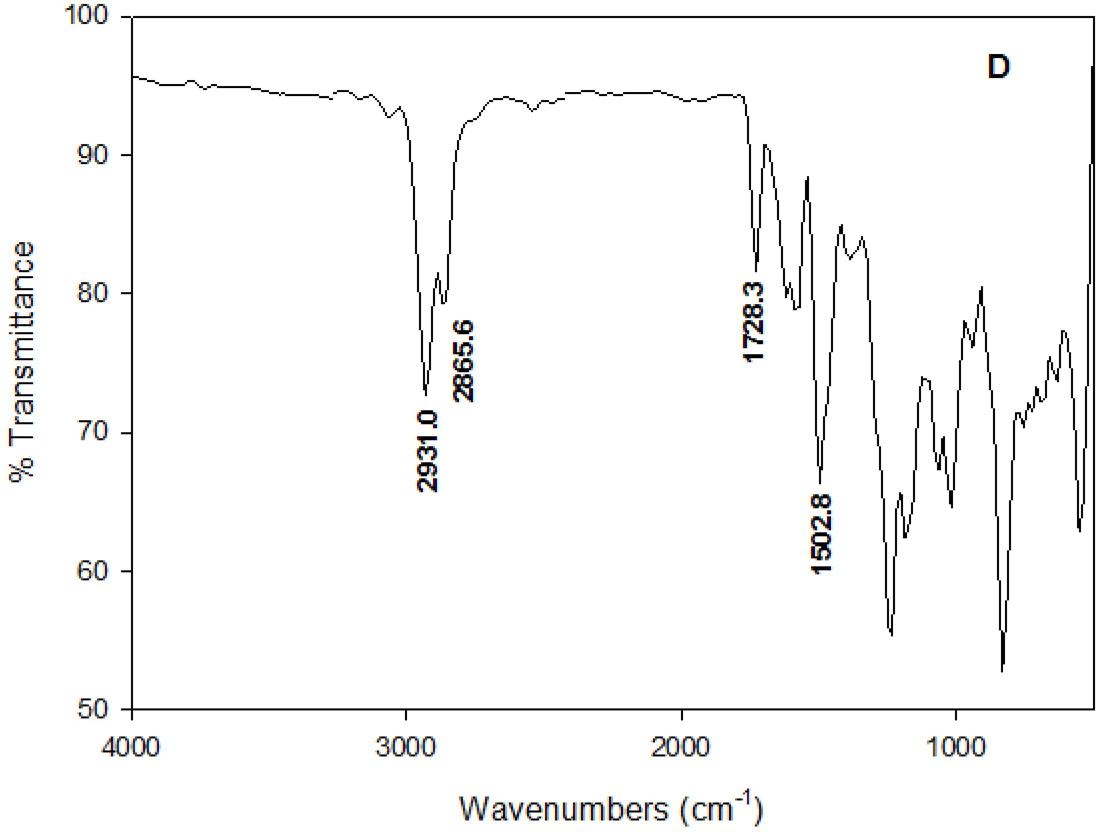

Figure S11: FTIR of 4-fluorophenyl 4-[4-(hexyloxy)phenylimino)methyl]benzoate (**D**)

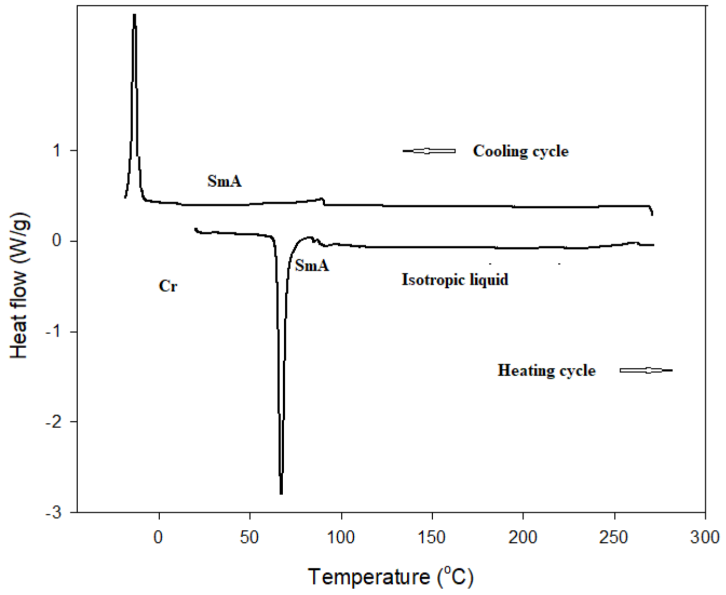

Figure S12: DSC thermograms of compound B upon heating and cooling cycles at heating rate 10 oC /min.
